# Immunohistochemical detection of high-mobility group box 1 correlates with resistance of preoperative chemoradiotherapy for lower rectal cancer: a retrospective study

**DOI:** 10.1186/1477-7819-13-7

**Published:** 2015-01-27

**Authors:** Kumiko Hongo, Shinsuke Kazama, Nelson H Tsuno, Soichiro Ishihara, Eiji Sunami, Joji Kitayama, Toshiaki Watanabe

**Affiliations:** Department of Surgical Oncology, Graduate School of Medicine, The University of Tokyo, 7-3-1 Hongo, Bunkyo-ku, Tokyo, 113-8655 Japan; Department of Transfusion Medicine, Graduate School of Medicine, The University of Tokyo, 7-3-1 Hongo, Bunkyo-ku, Tokyo, 113-8655 Japan

**Keywords:** High-mobility group box 1 (HMGB1), Lower rectal cancer, Chemoradiotherapy, Immunohistochemistry

## Abstract

**Background:**

High-mobility group box 1 (HMGB1) is a nucleoprotein that is related to inflammation. It has been implicated in a variety of biologically important processes, including transcription, DNA repair, differentiation, development, and extracellular signaling. Recently, its important role in the process of tumor invasion, metastasis, and resistance to anti-cancer therapies has been demonstrated. In this study, we aimed to investigate the correlation of HMGB1 expression and resistance of rectal cancer patients to chemoradiotherapy (CRT) prior to curative operation.

**Methods:**

We retrospectively reviewed the data of 75 lower rectal cancer patients without complete pathological response who had received preoperative CRT and had undergone curative resection at the University of Tokyo Hospital between May 2003 and June 2010. HMGB1 expression in surgically resected specimens was evaluated using immunohistochemical detection and specimens were classified into high or low HMGB1 expression groups. Clinicopathologic features, degree of tumor reduction, regression of tumor grade, and patient survival were compared between the groups using non-paired Student’s t-tests and Kaplan-Meier analysis.

**Results:**

A total of 52 (69.3%) patients had high HMGB1 expression, and 23 (30.7%) had low expression. HMGB1 expression was significantly correlated with histologic type (*P* = 0.02), lymphatic invasion (*P* = 0.02), and venous invasion (*P* = 0.05). Compared to patients with low HMGB1 expression, those with high expression had a poorer response to CRT, in terms of tumor reduction ratio (42.2 versus 28.9%, respectively; *P* <0.01) and post-CRT histological tumor regression grade (56.5 versus 30.8% grade 2; respectively; *P* = 0.03). However, no significant correlation was found between HMGB1 expression and recurrence-free and overall survival rates.

**Conclusions:**

HMGB1 expression may be one of the key factors regulating the response of rectal cancer to preoperative CRT in terms of tumor invasiveness and resistance to therapy.

## Background

High-mobility group box 1 (HMGB1) was first identified as a nuclear chromatin-binding protein that plays significant roles in various biologically important processes, including transcription, DNA repair, differentiation, and development [1]. In addition to its biological functions in the nuclear compartment, HMGB1 functions as an extracellular signaling molecule during inflammation, cell differentiation, cell migration, and metastasis [2–6]. HMGB1 is reported to be actively secreted by inflammatory cells when stimulated by endotoxin, tumor necrosis factor-α (TNF-α), or interleukin-1β (IL-1β), and is also passively released from necrotic cells [7, 8]. HMGB1 promotes inflammation by binding to the receptors, such as the receptor for advanced glycation end-products (RAGE), Toll-like receptor (TLR) 2, and TLR-4, which are expressed in a variety of cells including monocytes, macrophages, and endothelial cells [7, 9–12]. Through these actions, HMGB1 has been implicated in the pathogenesis of various clinical conditions, including sepsis [13], ischemia-reperfusion [14], meningitis [15], neurodegeneration [16], aging [17], and cancer [5, 6, 18].

In the pathophysiology of cancer, increased expression of HMGB1 and RAGE is associated with proliferative activity and metastatic potential in many types of tumors, including breast cancer [19], hepatocellular carcinoma [8], melanoma [20], glioma [21, 22], prostate cancer [23], gastric cancer [24], and colorectal cancer [25–27]. Increased RAGE-HMGB1 activity induces phosphorylation of extracellular signal-related kinase (ERK) [28], activating GTPases of the Rho family [29]. Thus, it contributes to cancer development through different mechanisms, including angiogenesis [28], cell migration [30], and apoptosis inhibition [24]. Moreover, extracellular reducible HMGB1 has been shown to induce autophagy and promote tumor resistance to alkylators, tubulin disrupting agents, DNA cross-linkers, and DNA intercalators in human pancreatic cancer and colon cancer cell lines [31]. Chemotherapy-induced HMGB1 expression in osteosarcoma cells promotes autophagy to inhibit apoptosis and increase drug resistance [32]. However, HMGB1 has a paradoxical dual effect on tumors [6]. HMGB1 has been shown to stimulate mature dendritic cells to degrade tumor antigen processing through its interaction with TLR-4 [33]. Furthermore, it mediates endogenous TLR-2 activation, resulting in tumor regression [34]. Therefore, the role of HMGB1 in cancer development and progression, as well as its effect on the response to treatment, remains largely unexplored.

Colorectal cancer is a highly invasive and metastatic tumor, and mortality associated with this cancer has increased worldwide recently [35]. Surgical resection and combined modality therapy, including chemotherapy, radiotherapy, and chemoradiotherapy (CRT), are the main therapeutic strategies for the management of rectal cancer. However, the effectiveness of these therapies greatly varies among patients, and those with a weaker response have a worse prognosis. In particular, those who do not respond to neoadjuvant CRT have a poor prognosis [36]. Thus, analysis of the molecular mechanisms underlying the resistance of rectal cancer cells to CRT is essential for the development of novel treatment strategies for the disease. In this study, we performed immunohistochemical analyses to examine HMGB1 expression in surgically resected specimens of rectal cancer after preoperative CRT and investigated its association with clinicopathological features, in an attempt to elucidate the possible association between HMGB1 expression and resistance to CRT.

## Methods

### Patients and evaluation of response to chemoradiotherapy

A total of 82 patients with lower rectal cancer who had received preoperative CRT and undergone curative resection at the University of Tokyo Hospital between May 2003 and June 2010 were enrolled. Patients receiving CRT had cancer in the middle or lower part of the rectum, with tumor invading further than the muscularis propria. CRT consisted of radiotherapy (1.8 Gy × 28 fractions = 50.4 Gy irradiation) and chemotherapy with a 5-fluorouracil (FU) prodrug (300 mg/m^2^/day) and leucovorin (75 mg/day), administered orally during the entire course of radiotherapy. We excluded seven patients with complete pathological response after CRT because in this study, we aimed to evaluate the residual cancer cells by immunohistochemical staining. Thus, among the 82 patients, 75 were considered eligible, and HMGB1 expression in surgically resected specimens was evaluated using immunohistochemical analyses. Clinicopathological features were analyzed on the basis of the TNM Classification of Malignant Tumors, Seventh edition, using the International Union Against Cancer (UICC) [37] and World Health Organization (WHO) histological criteria [38]. Post-CRT histological tumor regression was graded according to the seventh edition of the Japanese Guidelines for Clinical and Pathological Studies on Carcinoma of the Colorectum (Table 1) [39]. In this study, both grade 1a and 1b were classified together as grade 1. The reduction ratio was calculated based on the results of barium enema X-ray examination performed before and after CRT. The largest dimension of the tumor, from the same angle, before, and after CRT, was measured, and the post- to pre-CRT ratio was calculated. Patients’ consent and approval of the Ethics Committee of the University of Tokyo were obtained for the use of clinical samples for research purposes.

**Table 1:** **Grade of tumor regression after chemoradiotherapy for rectal carcinoma**

Grade		Tumor regression
Grade 0		Neither necrosis nor regressive change
Grade 1	a	>2/3 vital residual tumor cells
	b	Approximately 1/3 to 2/3 vital residual tumor cells
Grade 2		<1/3 vital residual tumor cells
Grade 3		No vital residual tumor cells

### Immunohistochemical evaluation

**Figure 1:**
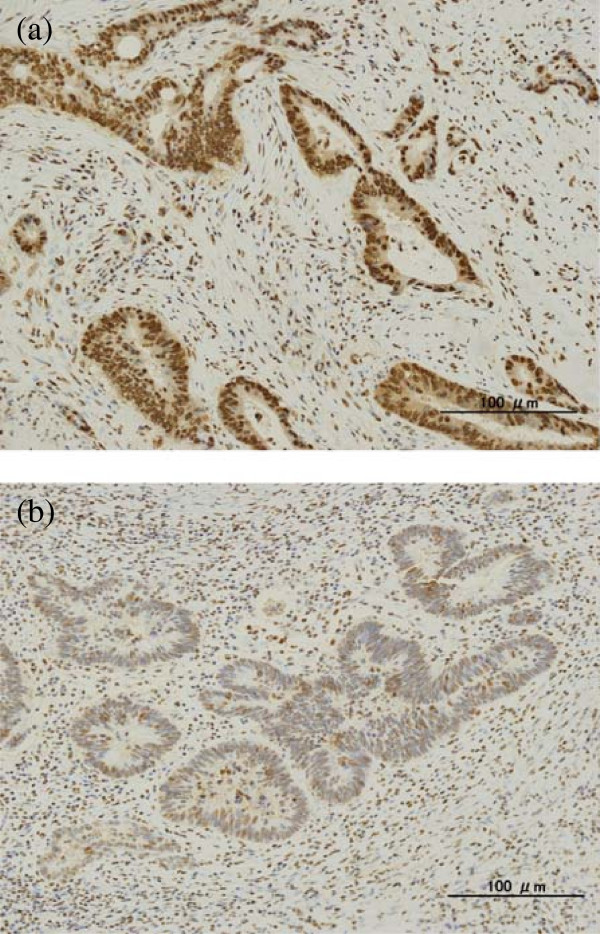
**HMGB1 Immunohistochemical staining of lower rectal carcinoma treated with chemoradiotherapy. (a)** High HMGB1 expression (original magnification, ×200). HMGB1 is predominant in the nuclei of tumor cells, and shows diffuse and strong expression. **(b)** Tumors with low HMGB1 expression (original magnification, ×200). HMGB1 expression is observed in focal tumor cells.

Consecutive formalin-fixed paraffin-embedded 4-μm sections were used for the immunohistochemical evaluation. After treatment with xylene and ethanol, followed by washing with phosphate-buffered saline (PBS), tumor specimens were subjected to heat-induced antigen retrieval in citrate buffer (Muto Pure Chemicals Co., Ltd, Tokyo, Japan). After washing with PBS, endogenous peroxidase was blocked with 3% hydrogen peroxide solution in methanol for 15 minutes (Junsei Chemical Co.Ltd, Tokyo, Japan). The tissues were then washed with PBS and were incubated with 5% bovine serum albumin (BSA) (Sigma Aldrich Chemical Co., St. Louis, Missouri, United States) for 30 minutes to block nonspecific antibody binding. The slides were then incubated overnight at 4°C with monoclonal antibodies against HMGB1 (Sigma Aldrich Chemical Co., St. Louis, Missouri, United States) at a dilution of 1:300. After washing three times with PBS, and incubation with biotinylated rabbit anti-mouse immunoglobulin-labeled globulin (Nichirei, Tokyo, Japan) for 20 minutes, Meyer’s hematoxylin (Sigma Aldrich Chemical Co., St. Louis, Missouri, United States) was used for counterstaining. One field per specimen, from an optimally stained area at a magnification of × 400, was randomly selected for evaluation. HMGB1 expression was strongly and predominantly detected in the nuclei of cancer cells, and it was also weakly observed in the cytoplasm of a few cases (Figure 1). Specimens were classified into high or low HMGB1 expression groups, according to their expression in residual cancer cells. When diffuse HMGB1 staining in the nuclei of residual cancer cells was observed, it was considered as high HMGB1 expression (Figure 1a), and when staining was only observed focally, it was considered as low expression (Figure 1b). Staining was evaluated independently by two observers trained in pathology (KH and SK) who were unaware of the clinical findings. Discrepancies between their findings were resolved by discussion. The correlations between HMGB1 expression and clinicopathological features, tumor recurrence-free survival, and overall survival rates were analyzed.

### Statistical analysis

The statistical significance of differences was evaluated using non-paired Student’s t-test, as appropriate. An association was considered significant when the exact significance level of the test was less than 0.05. Actuarial overall survival and recurrence-free rates were analyzed using the Kaplan-Meier method. The significance of several variables of the tumor regression grade after CRT was analyzed using logistic regression analysis in multivariate analysis.

## Results

### Patients’ characteristics

The clinicopathological findings of the 75 patients with lower rectal cancer who had received preoperative CRT and undergone surgical resection are listed in Table 2. A total of 45 patients (60.0%) were male, and 30 (40.0%) were female, with an age range of 33 to 79 years (mean 61.4 ± 10.2 years). There were 23 (30.7%) patients with stage 1 lower rectal cancer, 32 (42.7%) with stage 2, 13 (17.4%) with stage 3, and 7 (9.3%) with stage 4. According to the tumor regression grade, 46 (61.3%) were grade 1 and 29 (38.7%) were grade 2. The average reduction ratio after CRT was 33.0 ± 16.1%.

**Table 2:** **Characteristics of rectal cancer patients in this study**

Characteristics		
Age: mean age (years) ± SD		61.4 ± 10.2
Gender	M	45 (60.0%)
	F	30 (40.0%)
Tumor size (mm) ± SE		45.4 ± 8.6
Depth of tumor*	T1	7 (9.3%)
	T2	21 (28.0%)
	T3	42 (56.0%)
	T4	5 (6.7%)
Histologic type**	Well	49 (65.3%)
	Moderate	24 (32.0%)
	Poor	0 (0%)
	Mucinous	2 (2.7%)
Lymphatic invasion	Positive	7 (9.3%)
	Negative	68 (90.7%)
Venous invasion	Positive	39 (52.0%)
	Negative	36 (48.0%)
Lymph node metastasis	Positive	15 (20.0%)
	Negative	60 (80.0%)
Distant metastasis	Positive	2 (1.3%)
	Negative	152 (98.7%)
Liver metastasis	Positive	1 (0.6%)
	Negative	153 (99.4%)
Stage*	1	23 (30.7%)
	2	32 (42.7%)
	3	13 (17.4%)
	4	7 (9.3%)
Tumor regression grade	1	46 (61.3%)
	2	29 (38.7%)
Reduction ratio (%) ± SD		33.0 ± 16.1

*TNM Classification of Malignant Tumors, seventh edition. **World Health Organization (WHO) histological criteria.

### Correlation between clinicopathological features and HMGB1 expression in rectal cancer patients treated with chemoradiotherapy

The correlation between HMGB1 expression and the clinicopathological characteristics of tumors is shown in Table 3. A total of 52 (69.3%) patients had high HMGB1 expression, and 23 (30.7%) had low expression. No correlation was found between HMGB1 expression and age, gender, tumor size, depth of tumor, lymph node metastasis, and TNM stage. However, HMGB1 expression significantly correlated with the histological type of the tumor (*P* = 0.02), lymphatic invasion (*P* = 0.02), and venous invasion (*P* = 0.05). Well-differentiated tumors were observed in 87% (20 out of 23) of tumors with low HMGB1 expression and 55.8% (29 out of 52) of tumors with high expression (*P* = 0.02). Lymphatic invasion was identified in 13.5% (seven out of 52) of tumors with high HMGB1 expression, but in no cases with low HMGB1 (*P* = 0.02). Venous invasion was present in 59.6% (31 out of 52) of tumors with high HMGB1 expression compared to 34.8% (eight out of 23) of those with low expression (*P* = 0.05). Moreover, compared to low expression, high HMGB1 expression was associated with a poorer response to CRT, in terms of both the tumor reduction ratio (42.2 versus 28.9%, respectively; *P* <0.01) and the post-CRT histological tumor regression grade (43.5 versus 69.2% and 56.5 versus 30.8%, respectively for grades 1 and 2; *P* = 0.03).

**Table 3:** **Correlation between the clinicopathologic features and HMGB1 expression**

Characteristics		HMGB1	HMGB1	***P*** value
		High expression	Low expression	
		52 (69.3%)	23 (30.7%)	
Age: mean ± SD (years)		61.6 ± 10.3	61.1 ± 10.2	0.87
Gender	M	30 (57.7%)	15 (65.2%)	0.54
	F	22 (42.3%)	8 (34.8%)	
Tumor size (mm) ± SE		5.35 ± 1.02	2.72 ± 1.53	0.16
Depth of tumor*	T1	5 (9.6%)	2 (8.7%)	0.55
	T2	12 (23.1%)	9 (39.1%)	
	T3	31 (59.6%)	11 (47.8%)	
	T4	4 (7.7%)	1 (4.4%)	
Lymph node metastasis	Positive	13 (25.0%)	2 (8.7%)	0.08
	Negative	39 (75.0%)	21 (91.3%)	
Histologic type**	Well	29 (55.8%)	20 (87.0%)	0.02
	Moderate	21 (40.4%)	3 (13.0%)	
	Mucinous	2 (3.9%)	0 (0%)	
Lymphatic invasion	Positive	7 (13.5%)	0 (0%)	0.02
	Negative	45 (86.5%)	23 (100%)	
Venous invasion	Positive	31 (59.6%)	8 (34.8%)	0.05
	Negative	21 (40.4%)	15 (65.2%)	
Tumor regression grade	1	36 (69.2%)	10 (43.5%)	0.03
	2	16 (30.8%)	13 (56.5%)	
Reduction ratio (%) ± SD		28.9 ± 2.1	42.2 ± 3.1	0
Stage**	1	14 (26.9%)	9 (39.1%)	0.22
	2	21 (40.4%)	11 (47.8%)	
	3	11 (21.1%)	2 (8.7%)	
	4	6 (11.5%)	1 (4.4%)	

*TNM Classification of Malignant Tumors, seventh edition. **World Health Organization (WHO) histological criteria.

### Recurrence-free survival and overall survival analysis of rectal cancer patients in relation to HMGB1 expression

**Figure 2:**
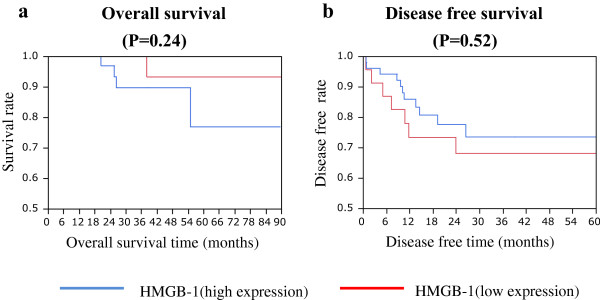
**Recurrence-free survival and overall survival analysis of colon cancer patients in relation to HMGB-1 expression. (a)** Overall survival rates. **(b)** Kaplan-Meier plot showing disease-free survival rates of rectal cancer patients receiving preoperative chemoradiotherapy.

Using Kaplan-Meier analysis, the log-rank test revealed no significant correlation between the expression of HMGB1 and recurrence-free survival, overall survival (Figure 2a, b), and local recurrence (data not shown).

## Discussion

HMGB1 is both a nuclear factor and a secreted protein that acts as a damage-associated molecular pattern molecule (DAMP) [40]. Recently, HMGB1 has been shown to play an important role in cancer biology, including angiogenesis, apoptosis, growth signals, tissue invasion, and metastasis [41–43]. In this study, we focused on the HMGB1 staining pattern in the nuclear compartment, but not the cytoplasm, of rectal cancer cells, and evaluated its possible role in tumor progression and resistance to treatment.

In our series, HMGB1 expression significantly correlated with the histological type of tumor, lymphatic invasion, and venous invasion in rectal cancer. Patients with high HMGB1 expression were more resistant to CRT, as revealed by lower tumor reduction ratio and lower post-CRT histological tumor regression grade. This may imply that rectal cancers with high HMGB1 expression have higher malignancy potential, acquiring resistance to CRT. Corroborating these findings, we observed that HMGB1 expression significantly correlated with lymphatic and venous invasion. In oral cancer, HMGB1 has been reported to promote lymphangiogenesis through the upregulation of vascular endothelial growth factor C (VEGF-C) and vascular endothelial growth factor D (VEGF-D) [44], which might be linked to the transmigration of HMGB1 [27]. Furthermore, in nasopharyngeal carcinoma cells, endogenous HMGB1 expression was associated with invasiveness [41].

Possible mechanisms of induction of resistance to radiotherapy and chemotherapy by HMGB1 may be: 1) the facilitation of protein-protein interaction and recognition of DNA damage in the process of mismatch repair [45]; 2) regulation of autophagy [31]; and 3) regulation of heat shock protein beta-1 (HSPB1) gene expression, which regulates mitophagy [46]. After DNA damage induced by ultraviolet light irradiation or platination, HMGB1 is sequestered in the nucleus, which is classically associated with apoptosis [8]. An increased level of HMGB1 might promote DNA repair induced by radiation. In addition, HMGB1 is a critical regulator of autophagy [31], promoting drug resistance in osteosarcoma [32] and leukemia cells [47]. Chemotherapy-induced HMGB1 expression in osteosarcoma cells promoted autophagy through controlling the formation of the Beclin 1-phosphatidylinositol 3-kinase class 3 (PI3KC3) complex to inhibit apoptosis and increase drug resistance [32]. Most cancer therapies, such as radiation and anti-cancer drugs, induce cancer cells to undergo autophagy [48, 49], which is an important mechanism of resistance to therapy; thus, mechanisms involving HMGB1 might be key regulators of resistance to anti-cancer therapies. Furthermore, nuclear HMGB1 regulates HSPB1 gene expression. Mitophagy is responsible for the elimination of dysfunctional and impaired mitochondria. It is unclear whether or how mitophagy triggered by dysfunctional mitochondria is regulated by nuclear mediators. However, it has been recognized that HMGB1 modulates mitochondrial respiration and morphological features by helping to sustain autophagy in mitochondrial maintenance through regulation of HSPB1 gene expression [46]. Autophagy and mitophagy, therefore, are involved in sustaining mitochondrial respiration and morphological features after cellular stress and mitochondrial injury. These mechanisms of nuclear HMGB1, including DNA repair, and regulation of autophagy and mitophagy, might be involved in the development of resistance of rectal cancer cells to CRT. Thus, targeting of HMGB1 may be a promising approach for the development of novel therapeutic strategies for rectal cancer.

In our series, however, no significant correlation between HMGB1 expression and recurrence-free or overall survival was found. Since HMGB1 is reported to have paradoxical effects on tumor progression by affecting both the cancer cells and the tumor immunity, the counter-balance between these two effects may be important in determining the final effect. However, caution is required in interpreting our present data. HMGB1 could not be identified as an effective predictive factor for CRT, because only tumor tissues obtained after CRT were used for immunohistochemical staining of HMGB1, and immunostaining of HMGB1 may be affected by CRT itself. Another limitation was the small number of patients, and the retrospective nature of the study.

## Conclusions

In conclusion, using immunohistochemistry, this study demonstrated the association of HMGB1 expression in human rectal cancer tissue exposed to CRT with tumor invasiveness and resistance to therapy. HMGB1, which regulates both cell death and cell survival, likely plays a role in the development of carcinogenesis and chemoresistance. Further large-scale prospective studies with long-term follow-up periods, evaluating samples obtained pre- and post-CRT, are needed to determine the potential role of HMGB1 as a prognostic factor for CRT in rectal cancer.

## Abbreviations

CRT: Chemoradiotherapy
HMGB1: High-mobility group box 1
HSPB1: Heat shock protein beta-1
RAGE: Receptors such as advanced glycation end-products
TLR-2: Toll-like receptor 2
TLR-4: Toll-like receptor 4
DAMP: damage associated molecular pattern molecule
VEGF-C: vascular endothelial growth factor C
VEGF-D: vascular endothelial growth factor D
HSPB1: heat shock protein beta-1
PI3KC3: phosphatidylinositol 3-kinase class 3.
